# Development and Validation of an Artificial Intelligence Model for Detecting Rib Fractures on Chest Radiographs

**DOI:** 10.3390/jcm13133850

**Published:** 2024-06-30

**Authors:** Kaehong Lee, Sunhee Lee, Ji Soo Kwak, Heechan Park, Hoonji Oh, Jae Chul Koh

**Affiliations:** 1 Department of Anesthesiology and Pain Medicine, Korea University College of Medicine, Seoul 02841, Republic of Korea; carrot809@korea.ac.kr (K.L.); sunfanooo@kumc.or.kr (S.L.); calorie@korea.ac.kr (J.S.K.); qgc9461@korea.ac.kr (H.P.); 2 Department of Biostatistics, College of Medicine, Korea University, Seoul 02841, Republic of Korea; hchavi531@korea.ac.kr

**Keywords:** artificial intelligence (AI), rib fractures, chest radiograph, rib fracture AI model, rib fracture detection, rib fracture localization, radiograph classification, convolutional neural network (CNN), deep learning model, Detectron2

## Abstract

**Background**: Chest radiography is the standard method for detecting rib fractures. Our study aims to develop an artificial intelligence (AI) model that, with only a relatively small amount of training data, can identify rib fractures on chest radiographs and accurately mark their precise locations, thereby achieving a diagnostic accuracy comparable to that of medical professionals. **Methods**: For this retrospective study, we developed an AI model using 540 chest radiographs (270 normal and 270 with rib fractures) labeled for use with Detectron2 which incorporates a faster region-based convolutional neural network (R-CNN) enhanced with a feature pyramid network (FPN). The model’s ability to classify radiographs and detect rib fractures was assessed. Furthermore, we compared the model’s performance to that of 12 physicians, including six board-certified anesthesiologists and six residents, through an observer performance test. **Results**: Regarding the radiographic classification performance of the AI model, the sensitivity, specificity, and area under the receiver operating characteristic curve (AUROC) were 0.87, 0.83, and 0.89, respectively. In terms of rib fracture detection performance, the sensitivity, false-positive rate, and free-response receiver operating characteristic (JAFROC) figure of merit (FOM) were 0.62, 0.3, and 0.76, respectively. The AI model showed no statistically significant difference in the observer performance test compared to 11 of 12 and 10 of 12 physicians, respectively. **Conclusions**: We developed an AI model trained on a limited dataset that demonstrated a rib fracture classification and detection performance comparable to that of an experienced physician.

## 1. Introduction

Rib fractures are among the most frequently encountered injuries by trauma teams, operating room staff, and pain medicine clinics, highlighting the need for effective diagnostic techniques. They are the most common thoracic injuries, occurring in 10% of all traumatic injuries and in almost 40% of patients who have suffered severe injuries or blunt chest trauma [1]. Chest radiography and computed tomography (CT) are the two main modalities used to detect rib fractures [2,3]. Although there are more accurate methods for diagnosing rib fractures, such as CT, thoracic CT can expose patients to radiation equivalent to 50–450 chest radiographs [4]. Moreover, because of the cost and time required, CT is not universally administered, especially in patients with minor blunt trauma. Conversely, chest radiography, which has a relatively lower sensitivity, is a routine examination that offers significant economic and clinical advantages beyond health screening [5,6]. The American College of Radiology recommends plain chest radiography as the initial test for suspected rib fractures after minor trauma, establishing it as the first-line imaging modality [2]. However, up to 50% of rib fractures may be missed on standard X-rays [7]. The low detection rate may be attributable to various factors. First, rib fractures can be identified incidentally on chest radiography. Among the various diagnoses detectable on chest radiography, the identification of rib fractures can be challenging for clinicians. Rib fractures may not be easily suspected in patients with communication difficulties, such as older individuals or those with cerebrovascular disease. Additionally, the ambiguity resulting from overlapping anatomical structures, as well as the varying locations and sizes of fractures on radiographs, contributes to this challenge [8]. However, image interpretation requires considerable experience and training. While ultrasound can be helpful, it requires applying pressure with the probe on suspected rib fractures, can produce artifacts, demands operator proficiency, and is time-consuming, which limits its use [9]. Therefore, developing a rapid, accurate, and accessible automatic detection system for rib fractures on chest radiographs is crucial for improving diagnostic precision, assisting clinicians, and establishing cost-effective tools.

Advances in artificial intelligence (AI) and automatic medical image processing technologies have opened promising avenues for development and innovation in various medical fields [10,11]. Research has been conducted on the development of AI to analyze medical images using neural networks, which are mostly composed of convolutional neural networks. In these studies, attempts were made to develop algorithms with better performance by combining layers of different configurations. Through this process, algorithms that exhibit excellent performance in most image analyses have been introduced [12,13,14]. However, it is difficult to analyze medical images of various resolutions and present the desired results with a combination of these neural networks alone. Therefore, it has become important to use an algorithm that can automatically change images that exist in various forms into a form suitable for application in neural networks.

The purpose of our study was to develop an AI platform specifically designed to detect rib fractures on chest radiographs, implement an intuitive interface for clinician review, and validate its detection performance with that of experienced clinicians.

## 2. Materials and Methods

### 2.1. Dataset Preparation

**Figure 1:**
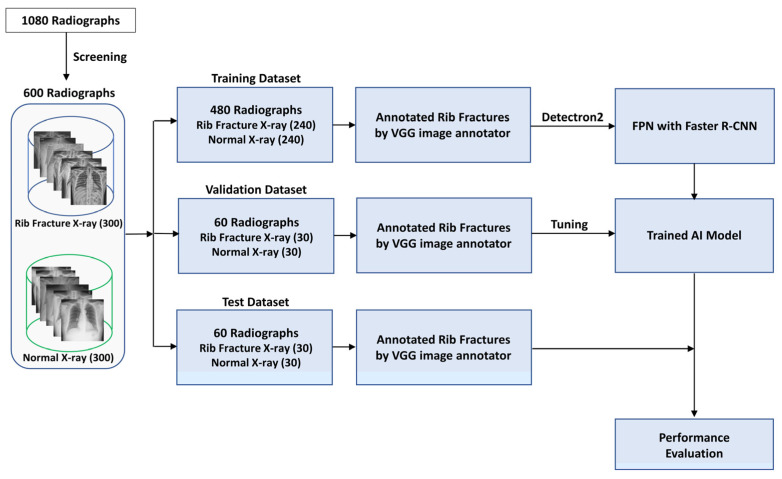
Study flowchart for the development and evaluation of an artificial intelligence model for rib fracture detection and localization on chest radiographs. AI = Artificial Intelligence, VGG = Visual Geometry Group, Faster R-CNN = Faster Region-Convolutional Neural Network.

For the AI model development, 1080 chest antero-posterior or posteroanterior view radiographs with rib fracture findings based on radiology reports were retrospectively collected between January 2017 and November 2023 from the anonymized picture archiving and communication system (PACS) database at our institution (Korea University Anam Hospital) (Figure 1). Chest radiographs were excluded from the training dataset if rib fractures were not distinctly identifiable even after verification with the associated CT scans, if the image quality was compromised due to factors such as underexposure, motion blur, or insufficient resolution, or if the radiographs were duplicates of cases already included. After these exclusions, the dataset included 300 chest radiographs of patients with rib fractures. To ensure comparability, we matched these with 300 normal chest radiographs, selecting images from 300 different patients who had no recorded abnormalities in their radiology reports during the same period, using the PACS system. Subsequently, the chest radiographic data were randomly allocated to one of three distinct datasets to assess the classification and detection performance of the trained AI model: a training dataset consisting of 480 chest radiographs (240 normal and 240 with rib fractures), a validation dataset containing 60 chest radiographs (30 normal and 30 with rib fractures), and a test dataset containing 60 chest radiographs (30 normal and 30 with rib fractures). The training dataset included 240 chest radiographs of 771 rib fractures, the validation dataset comprised 30 chest radiographs of 77 rib fractures, and the test dataset contained 30 chest radiographs of 73 rib fractures. The validation dataset was used to assess model performance and fine-tune hyperparameters during the training process to prevent overfitting before applying the model to unseen data. Conversely, the test dataset was used to evaluate the performance of the final model after optimization of these hyperparameters. Additionally, an observer performance test using the same test dataset was conducted to compare the performances. All chest radiographs in each dataset were obtained from different patients to ensure exclusivity among datasets.

### 2.2. Rib Fracture Annotation on Chest Radiographs

**Figure 2:**
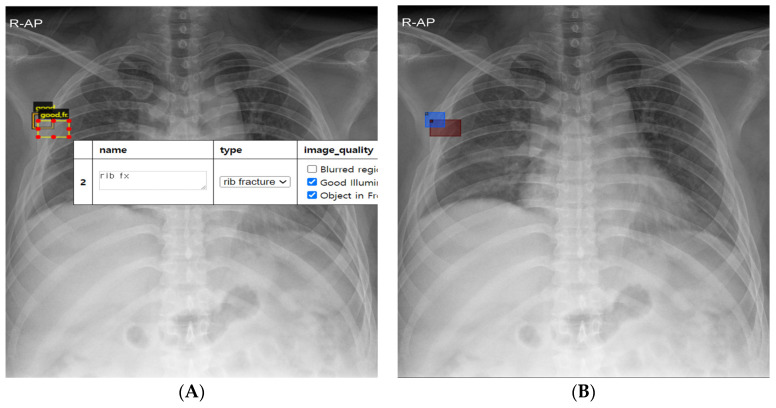
Annotation and preprocessing of rib fracture radiographs for AI training (**A**) Annotated rib fractures placed in the middle of the bounding box using Visual Geometry Group (VGG) annotator. (**B**) Converted region of interest as a bounding box to be prepared for artificial intelligence training.

Chest radiographs from the training, validation, and test datasets were labeled as either normal or rib fracture at the image level, and the location of rib fractures on rib fracture chest radiographs was annotated at the pixel level (Figure 2A). The labeling for each radiograph and annotation for each rib fracture were performed based on interpretations by radiologists using the basic annotator (https://www.robots.ox.ac.uk/~vgg/software/via/via_demo.html (accessed on 17 October 2022)) provided by the Visual Geometry Group (University of Oxford, Department of Engineering). The pixel area containing rib fracture lesions was annotated by placing it at the center of the bounding box, ensuring that it was minimized in size yet sufficient to fully encompass both cross-sections of the fractured rib. Chest CT scans and rib series radiographs were used as reference standards, and annotations were based on comparisons with these imaging modalities as well as radiology reports. Annotations included up to seven multiple rib fractures per chest radiograph, encompassing clear displacement cases, non-displacement cases with intact alignment, and rib fractures in a healing state characterized by the presence of bony calluses.

### 2.3. Development of AI Model

After completing labeling and annotation, the generated file included the coordinates of the center point and the horizontal and vertical dimensions of the rectangle around the rib fractures. To translate this information into a trainable format, we created a program that automatically designated each annotated rectangle as a bounding box and the region within it for segmentation in preparation for training (Figure 2B).

Detectron2, an open-source platform created by Facebook AI Research (FAIR), was used for model development [15]. This platform efficiently combines neural networks, feature pyramid networks, and region proposal networks, enabling superior performance with less training data compared to previous medical AI developments. Based on this library, we applied the Faster_R-CNN_R_101_FPN_3x model to develop the AI model. This architecture utilizes preconfigured Faster Regions with Convolutional Neural Networks (Faster R-CNN) by applying a ResNet-101 backbone and a Feature Pyramid Network (FPN) [16]. To optimize the model, hyperparameters were fine-tuned during the training process as follows: the batch size was set to 512, the base learning rate was set to 0.00025, and 4 images per batch were used up to 30,000 iterations. We adopted this platform and programmed it so that the transformed trainable data mentioned above could be subsequently applied.

**Figure 3:**
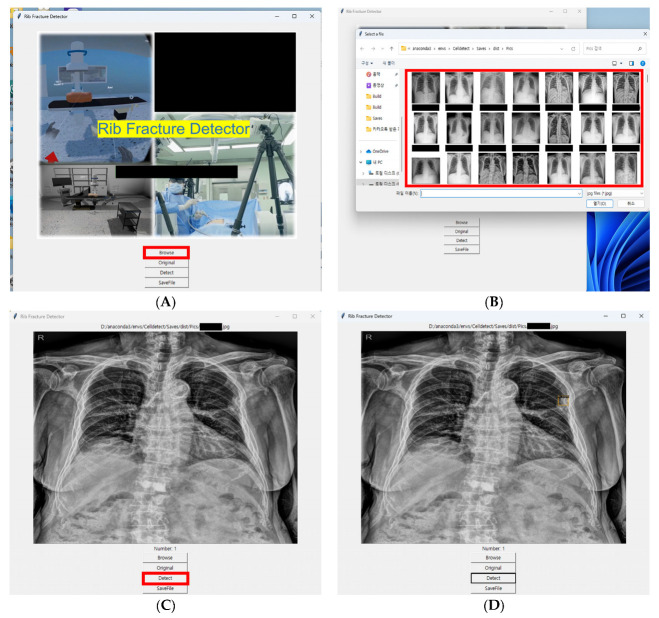
AI-powered rib fracture detection system interface and diagnostic output (**A**) The initial interface of the program is designed for convenient execution in a clinical setting. (**B**) A user-friendly interface is designed to facilitate easy selection of a file/image to be analyzed. (**C**) The original chest radiograph before the site of the fracture is identified. (**D**) The image with the site of the rib fracture identified and marked by the AI model. AI: Artificial Intelligence.

The Python Tkinter library was used to develop an executable program that can be easily applied in clinical settings. By creating this program, we built a system designed to operate seamlessly in real clinical environments. This system enables clinicians to capture X-ray images while seeing patients, which are then imported into the program as image files. This approach bypasses the need for direct integration with electronic health records. Additionally, we implemented an intuitive interface that allows clinicians to quickly review and verify the AI’s findings. Using a pre-trained model, the system promptly identifies and reports potential rib fractures (Figure 3).

### 2.4. Evaluation of AI Model

The AI model identified suspected rib fracture sites on radiography with a confidence level greater than 5%, marking these areas as potential fractures with boxes, and generating continuous numeric values between 0 and 1 alongside them to indicate the probability of a fracture. In cases in which multiple fracture probabilities were identified on a single chest radiograph, the highest value was designated as the fracture probability. Conversely, if no areas were marked, the chest radiograph showed a 0% probability of rib fracture. Radiograph classification and fracture detection performance were assessed on a per-case basis for the validation and test datasets, and on a per-fracture basis, respectively. An observer performance test was conducted using the test dataset to compare the radiographic classification and fracture detection performance of the AI model with those of physicians. Twelve physicians, divided into two subgroups (six board-certified anesthesiologists, specifically pain medicine specialists who are experienced in both radiographic interpretation and patient care, and six anesthesiology residents training in pain medicine), participated as observers. During the residency program, trainees receive comprehensive training in both fields, including the ability to interpret and analyze imaging studies for patients with various types of pain, such as diagnosing rib fractures. Each observer independently reviewed each chest radiograph to distinguish normal chest radiographs from those with rib fractures (radiograph classification) and to identify the location of rib fractures (fracture detection) using a four-staged confidence scale: 25% indicated a potential lesion with a low degree of suspicion, 50% indicated a dubious lesion, 75% indicated a probable lesion with high confidence, and 100% indicated a definite lesion.

### 2.5. Statistical Analysis

**Figure 4:**
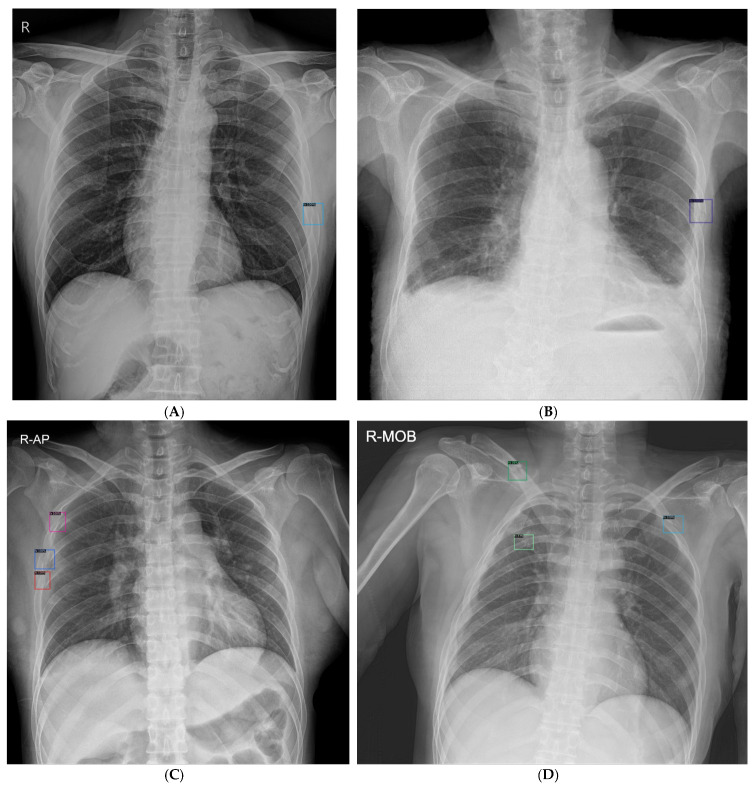
Examples of rib fractures detected on chest radiographs using the AI model (**A**) Single rib fracture: The AI model’s correct identification of a rib fracture is demonstrated on the left lateral side, marked with a predicted box and assigned a 100% probability of fracture. (**B**) Single rib fracture in low resolution shows the AI model’s capacity to precisely mark a rib fracture location on a low-resolution image. (**C**) Multiple rib fractures: This image displays the AI model’s marking of several rib fracture sites, with each predicted box accompanied by a respective estimated probability of fracture. (**D**) Accurate and erroneous marking: This image features the AI model’s accurate marking of a rib fracture on the left side and a false-positive marking on the right side. Additionally, it includes an unintended marking of a clavicle fracture, not originally part of the training data. AI: Artificial Intelligence.

The performance of the AI model in the radiograph classification task, which involves determining the presence or absence of rib fractures on each chest radiograph, was evaluated using the AUROC curve as a measure of probability. For the fracture detection task, which entailed localizing rib fracture lesions on a chest radiograph, the assessment was conducted using figures of merit (FOMs) obtained from the jackknife alternative free-response receiver operating characteristic analysis (JAFROC version 4.2.1; available at https://github.com/dpc10ster/WindowsJAFROC (accessed on 11 October 2023)). This method evaluates the diagnostic accuracy of imaging techniques by analyzing their efficacy in detecting and localizing abnormalities in images. In JAFROC, the FOM represents the likelihood that true lesions are rated higher than the highest-rated non-lesion mark on a normal chest radiograph, with higher values indicating better performance. A prediction was deemed accurate if there was an overlap between the predicted and annotated bounding boxes. A true positive was defined when the overlapping area between the annotated and predicted boxes exceeded 30%; otherwise, any predicted box marked outside this criterion was defined as a false positive. When the overlap between the annotated and predicted boxes was less than 30%, the fracture was classified as a false negative. Examples of rib fractures detected on chest radiographs using the AI model are shown in Figure 4.

Regarding the observer performance test, we compared the performance of our AI model with that of experienced clinicians by conducting a test where both the AI system and the clinicians were given the same test dataset of anonymized chest radiographs. The results were evaluated based on sensitivity, specificity, false positive ratio, and metrics such as the area under the receiver operating characteristic (AUROC) and the Free-response Receiver Operating Characteristic (FROC). The radiographic classification and rib fracture detection capabilities of the AI model and 12 physicians (six board-certified anesthesiologists and six anesthesiology residents) were assessed and compared using the pairwise comparison AUROC and JAFROC FOMs, respectively. To compare AUC between an individual observer and the AI model, DeLong’s test was conducted [17]. To compare JAFROC FOMs between an individual observer and the AI model, a fixed-reader random-case method was used [18]. The random-case, random-reader method was used to average the performances of the two subgroups: a board-certified anesthesiologist subgroup and an anesthesiology resident group. *p*-value < 0.05 was considered statistically significant.

## 3. Results

### 3.1. Training Iterations Experiment

**Figure 5:**
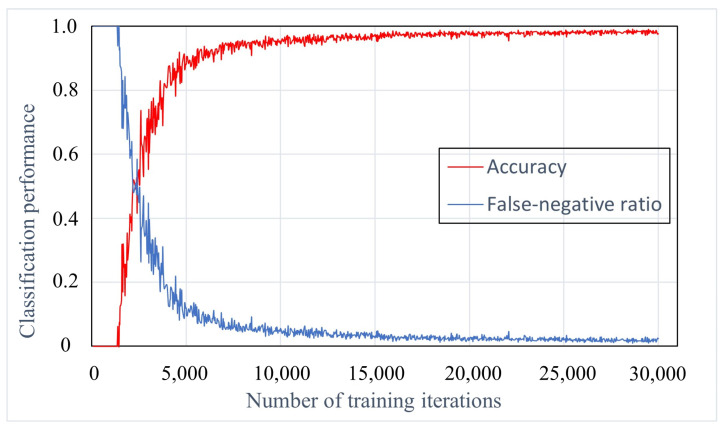
Classification performance of the AI model with machine learning training iterations. After reaching 10,000 training iterations, improvement in both the accuracy and the false-negative ratio tends to plateau.

To determine a sufficient number of learning iterations, we varied the number of training iterations in the dataset from 500 to 30,000 and then evaluated the performance of the developed AI model in terms of accuracy and false-negative ratio. When comparing the AI models developed with 10,000 and 30,000 learning iterations, there was only a 0.01 increase in the accuracy and a 0.01 decrease in the false-negative ratio (Figure 5).

### 3.2. Classification Performance of AI Model

The performance of the AI model is assessed using validation and test datasets. For the validation data set of 60 chest radiographs (30 normal and 30 with rib fractures), the sensitivity and specificity analyses resulted in values of 0.7 and 0.93, respectively. The AI model achieved an AUROC of 0.83. In the test dataset (30 normal subjects and 30 patients with rib fractures), the sensitivity, specificity, and AUROC were 0.87, 0.83, and 0.89, respectively (Table 1).

### 3.3. Rib Fracture Detection Performance of AI Model

For the validation dataset, the sensitivity and false-positive rate were 0.49 and 0.27, respectively. The JAFROC FOM was 0.72. In the test dataset, the sensitivity, specificity, and JAFROC FOM were 0.62, 0.3, and 0.76, respectively (Table 1).

### 3.4. Comparison of Radiograph Classification and Fracture Detection Performance between AI Model and Observers

In the radiographic classification, the sensitivity of the AI model (0.87) was equal to or higher than that of seven out of 12 physicians (three board-certified anesthesiologists and four anesthesiology residents). Additionally, the specificity of the AI model (0.83) matched or exceeded that of five out of 12 physicians (one board-certified anesthesiologist and four anesthesiology residents). In rib fracture detection, the sensitivity of the AI model (0.62) surpassed that of seven out of 12 physicians (two board-certified anesthesiologists and five anesthesiology residents). Moreover, the AI model’s false-positive rate (0.3) was lower than that of three out of 12 physicians (two board-certified anesthesiologists and one anesthesiology resident) (Table 2).

In assessing radiographic classification performance, the AI model demonstrated an AUROC of 0.89, which was higher than the results for five out of 12 physicians (one board-certified anesthesiologist and four anesthesiology residents). However, no statistically significant difference was found when compared with 11 of the 12 physicians (*p* > 0.05). Concerning rib fracture detection performance, the AI model exhibited a JAFROC FOM of 0.76, exceeding the performance of five out of 12 physicians (one board-certified anesthesiologist and four anesthesiology residents). Again, there was no statistically significant difference compared to 10 of the 12 physicians (*p* > 0.05). The JAFROC FOMs for the board-certified anesthesiologists and anesthesiology resident subgroups were 0.83 and 0.74, respectively. Random-reader analysis from JAFROC indicated that the difference in JAFROC FOM between the AI model and the group of six board-certified anesthesiologists was 0.07 (*p* = 0.051). The difference between the AI model’s JAFROC FOM and that of the six anesthesiology residents was 0.02 (*p* = 0.6) (Table 3).

## 4. Discussion

In this study, we developed an AI model to detect rib fractures on chest radiographs, achieving performance levels in chest radiograph classification and detection comparable to those of board-certified anesthesiologists and anesthesiology residents, even with a relatively small dataset.

CNNs have become a cornerstone in the field of AI applications within medical imaging, and their use is progressively expanding into the realm of medical diagnosis [19,20]. Studies have shown that multiple rib fractures are associated with significant morbidity and mortality particularly due to pulmonary complications [21]. This AI model can selectively classify chest radiographs with rib fractures with a sensitivity of 0.87 (26 out of 30), and an AUROC of 0.89. It also possesses multi-lesion detection capabilities, marking multiple rib fractures on chest radiographs with a sensitivity of 0.62 (45 out of 73) and a JAFROC of 0.76. However, traditional evaluations of radiographs have only been able to detect a small percentage of rib fractures, often less than 50% [22]. These results suggest that utilizing AI can enable clinicians to more effectively interpret chest radiographs alone, reducing the dependency on additional diagnostic procedures. Despite their high cost and increased patient exposure to radiation, advanced imaging is generally used, in cases with associated lesions of the thorax that have to be addressed or when there is a discrepancy between clinical findings and radiology. Plain X-rays remain the best method for detecting rib fractures [23].

The development of an AI model for diagnosing rib fractures was previously introduced [24]. Image classification studies were conducted to determine the presence or absence of rib fracture lesions on radiographs, and these image classification models can serve as screening tests to determine the necessity of more precise diagnostic examinations. However, it has been observed that doctors find it challenging to rely on these classification models, which do not indicate suspected areas but only suggest whether additional tests are recommended [25]. Typically, they prefer to directly confirm the basis of the AI judgment [26]. Meanwhile, the Faster R-CNN algorithm used in this study can identify a region of interest with a bounding box, along with classification, through a region proposal network. This detection allows clinicians to evaluate the soundness of the AI judgment and gain greater credibility from the results. Furthermore, the model may be useful for correlating patient symptoms with clinical outcomes, explaining them to patients, and establishing a treatment plan.

Training CT images have been used to create models that locate rib fracture sites [27,28,29]. However, chest CT is not used in routine practice because of its longer duration, higher economic costs, and potential risks associated with radiation exposure compared to plain chest radiography. It is commonly performed after diagnostic chest radiography. Therefore, the development of an AI model based on the widely used plain chest radiography would be more clinically useful. In particular, plain chest radiography is often used for basic screening purposes, allowing the detection of fractures, even in individuals who have difficulty communicating or are unconscious. Although fractures are easily missed, few attempts have been made to address this issue [30].

Recently, attempts have been made to diagnose rib fractures using chest radiography, as in this study [31,32,33]. Sun et al. [32] reported a sensitivity of 0.82 and an FROC of 0.87 with a false-positive count of 5 per image using their Fully Convolutional One-Stage system. However, when the false-positive count was reduced to 1, the sensitivity dramatically dropped to 0.60, and the FROC to 0.70. In comparison, our study achieved a sensitivity of 0.62 and a JAFROC FOM of 0.76, despite a much lower false-positive count of 0.3 per image. This result suggests that the lower false-positive count is a significant factor, especially considering that increasing sensitivity often requires sacrificing specificity. Huang et al. [31] achieved an accuracy range of 0.91–0.93 and an AUROC of 0.97–0.99 with their multi-CNN approach. However, their assessment used fixed pixel resolutions of 128 × 128, which differs from our study that evaluated performance using entire chest radiographs, precluding a direct comparison using FROC. Additionally, previous studies often involved image processing techniques that may not be practical in clinical settings. For example, Huang et al. employed data augmentation techniques such as flipping, rotation, and parallel shifting, and Wu et al. [33] standardized their images through multiscale image contrast amplification (MUSICA). In contrast, our study used unprocessed chest radiographs. Therefore, it is possible that our study’s observers applied more stringent criteria compared with previous studies. Furthermore, these studies required datasets ranging from several thousand to tens of thousands of images for effective training. In contrast, this study utilized a dataset of 540 images, which is relatively small compared with that of other studies. This is important because most medical settings may not have access to vast amounts of medical data. Hence, obtaining results that can be applied in clinical practice using only a limited dataset has meaningful implications. Selection of inputs and determination of expected outputs, even within a pre-established neural network architecture, are crucial for effective AI training. Simulating various data types is essential to ensure that clinicians can achieve the intended outcomes and make the necessary adjustments. We believe that the efficiency of our approach can be attributed to the involvement of a practicing clinician who tailored the AI training environment for optimization and closely monitored the training process to ensure its effectiveness. This AI training model is believed to be pivotal to achieving the desired efficiency without compromising the model performance in a clinical setting.

Previously introduced image analysis AI often required input images with a fixed resolution [33]. However, in this case, image downscaling is necessary to transform the image into a form for training artificial intelligence. When applying AI in clinical practice, models trained in this manner are often inconvenient. The chest radiographs used in clinical settings typically have different resolutions. If images with these various resolutions are not analyzed as they are and downscaling is performed, the quality of the images may deteriorate [34]. Additionally, performing this extra task in a busy clinical setting can be burdensome for clinicians. In this study, using an algorithm that integrates the ResNet-101-based Feature Pyramid Network into the program made it possible to optimize the training and detection performances while performing these processes conveniently and automatically [35].

The observer performance test showed that our AI model could detect rib fractures on chest radiographs with an accuracy comparable to that of physicians. Compared with a previous study that reported detection sensitivity with a false-positive rate of 1–5 per image [32], our AI model demonstrated a substantially decreased rate of false positives while preserving the AUROC and JAFROC values, similar to the performance of board-certified anesthesiologists. This significant reduction in false positives could lead to fewer unnecessary follow-up procedures, thereby minimizing patient exposure to radiation [36]. Although not assessed in this study, we postulate that providing observers with suspected rib fracture sites identified by AI could enhance diagnostic performance. This collaborative approach between AI and medical professionals is likely to further improve the diagnostic accuracy and efficiency of the clinical workflow [37].

To analyze images using AI, even after training, it is necessary to apply image data through various complex processes in a programming environment. However, it may present time and hardware challenges for use in the clinical environment [10]. Therefore, for convenience, a program should be created in such a way that it can be easily run on commonly used operating systems. However, the functions and interfaces that users require can differ from the developer’s configuration, which often causes inconvenience for users.

In the meantime, many developments in AI have been carried out by programmers specializing in AI rather than by users. However, in this study, all data preparation and training processes, including coding, were conducted directly by practicing clinicians. Therefore, we were able to develop a program that could be easily executed on Windows, which is a common operating system used on personal computers. Additionally, creating a program tailored to the needs of individual clinicians, which is convenient and efficient in clinical practice, is feasible. This was achieved by adopting a predetermined neural network architecture that has been previously developed and published. The use of predetermined neural networks offers several benefits to medical professionals. First, the time and effort required to construct a neural network can be reduced using an algorithm already optimized for image detection. Most image detection methods can perform well with a standardized algorithm without regenerating the neural network configuration. Even if a clinician has excellent coding skills, in most cases, sufficient time cannot be spent on coding because of the clinical work and the need to guide junior doctors. Second, the use of a predetermined neural network provides a standardized framework for image analysis, which can improve the consistency and reproducibility of the results across different medical centers and practitioners.

The demand for AI in healthcare is rapidly evolving and is seemingly endless. The AI model created in this study can be applied to any image analysis because it was performed according to the needs of clinicians, from training data generation to coding. Therefore, this model will be of great help in quickly meeting the increasing demand for AI in the medical field. Further advances in these technologies will help accelerate the advent of precision medicine, leading to pre-treatment simulations and intra-treatment guidance.

Our study has several limitations that should be considered. First, the relatively small dataset used for training may have impacted the accuracy of our rib fracture detection model. Additionally, the small validation sample size may have limited our ability to perform a detailed analysis of the diagnostic accuracy between physicians and the AI model. If we had validated interpretation skills against radiologists specializing in more advanced imaging interpretation, we might have obtained more objective and meaningful results. However, in this study, we consider that we have obtained meaningful data by comparing the interpretation abilities of AI with those of specialists who actively interpret imaging studies of real patients, as well as trainees who are training to interpret them. Moreover, if there had been comparisons with other AI models, this study could have produced more informative results. Lastly, the generalizability of our findings may be limited since the dataset was obtained from a single hospital. To improve the model’s applicability and robustness, future studies should incorporate datasets from multiple institutions into the AI training process.

## 5. Conclusions

We developed an AI model utilizing Detectron2 and trained on a limited dataset that achieved performances comparable to those of experienced physicians in radiograph classification and rib fracture detection.

## Figures and Tables

**Table 1:** Radiograph classification and rib fracture detection performance of the artificial intelligence (AI) model with the test dataset.

	Radiograph Classification Performance			Rib Fracture Detection Performance		
	AUROC	Sensitivity ^1^ (%)	Specificity (%)	JAFROC FOM	Sensitivity ^2^ (%)	Rate of FP ^3^ (%)
AI model	0.89	0.87 (26/30)	0.83 (25/30)	0.76	0.62 (45/73)	0.3 (18/60)

The data in parentheses are the numerators and denominators. AUROC = area under the receiver operating characteristic curve. JAFROC = jackknife alternative free-response receiver operating characteristic; FOM = figure of merit. ^1^ Sensitivity indicates the number of radiographs detected correctly divided by the number of radiographs showing fractures. ^2^ Sensitivity was calculated as the number of correctly detected rib fractures divided by the total number of rib fractures, for which the threshold value was set at 0.05. ^3^ The false-positive rate was calculated as the total number of fractures with false-positive findings divided by the total number of chest radiographs, for which the threshold value was set at 0.05.

**Table 2:** Classification and rib fracture detection performance of observers with the test dataset.

		Radiograph Classification Performance	Rib Fracture Detection Performance	
Observer ^1^	Sensitivity	Specificity	Sensitivity ^2^	Rate of FP ^3^
Board-certified anesthesiologists				
Observer 1	0.97 (29/30)	0.97 (29/30)	0.84 (61/73)	0.12 (7/60)
Observer 2	0.90 (27/30)	0.93 (28/30)	0.74 (54/73)	0.18 (11/60)
Observer 3	0.63 (19/30)	1.00 (30/30)	0.48 (35/73)	0.02 (2/60)
Observer 4	0.90 (27/30)	0.80 (24/30)	0.81 (59/73)	0.57 (34/60)
Observer 5	0.87 (26/30)	0.93 (28/30)	0.59 (43/73)	0.13 (8/60)
Observer 6	0.87 (26/30)	0.93 (28/30)	0.71 (52/73)	0.33 (20/60)
Anesthesiology residents				
Observer 7	0.77 (23/30)	0.93 (28/30)	0.59 (43/73)	0.17 (10/60)
Observer 8	0.90 (27/30)	0.80 (24/30)	0.70 (51/73)	0.35 (21/60)
Observer 9	0.93 (28/30)	0.70 (21/30)	0.58 (42/73)	0.25 (15/60)
Observer 10	0.87 (26/30)	0.77 (23/30)	0.55 (40/73)	0.20 (12/60)
Observer 11	0.87 (26/30)	0.77 (23/30)	0.56 (41/73)	0.25 (15/60)
Observer 12	0.87 (26/30)	0.90 (27/30)	0.60 (44/73)	0.22 (13/60)

Data in parentheses are the numerators and denominators. ^1^ Experience, Observer 1–6: 6 to 12 years of clinical experience after obtaining board, Observer 7–12: 1 to 4 years of residency. ^2^ Sensitivity was calculated as the number of correctly detected rib fractures divided by the total number of rib fractures, for which the threshold value was set at 0.05. ^3^ The false-positive rate was calculated as the total number of fractures with false-positive findings divided by the total number of chest radiographs, for which the threshold value was set at 0.05.

**Table 3:** Classification and rib fracture detection at the observer performance test.

	Test		AI Model versus Observer (*p* Value)	
	Radiograph Classification (AUROC)	Rib Fracture Detection (JAFROC FOM)	Radiograph Classification	Rib Fracture Detection
Board-certified anesthesiologists				
Observer 1	0.98	0.91	0.013	<0.001
Observer 2	0.95	0.86	0.054	0.028
Observer 3	0.82	0.74	0.2	0.7
Observer 4	0.90	0.85	0.9	0.050
Observer 5	0.93	0.78	0.4	0.6
Observer 6	0.93	0.85	0.4	0.059
Group		0.83		0.051
Anesthesiology residents				
Observer 7	0.87	0.73	0.7	0.6
Observer 8	0.90	0.84	0.8	0.093
Observer 9	0.88	0.68	0.9	0.079
Observer 10	0.88	0.70	0.9	0.2
Observer 11	0.88	0.71	0.8	0.3
Observer 12	0.92	0.78	0.4	0.6
Group		0.74		0.6
AI model	0.89	0.76		

Radiograph classification performance was described using the area under the receiver operating characteristic curve analysis, and associated *p*-values were calculated using comparison receiver operating characteristic analysis. Rib fracture detection performance was described using a figure of merit from jackknife free-response receiver operating characteristic analysis, and associated *p* values were calculated using the fixed readers and random cases method for the AI model for observer comparison using jackknife free-response receiver operating characteristic analysis. AI model, artificial intelligence model; AUROC, area under the receiver operating characteristic curve; JAFROC, jackknife alternative free-response receiver operating characteristic; FOM, figure of merit.

## Data Availability

The data presented in this study are available on request from the corresponding author.
